# Retropharyngeal abscess with mediastinal extension – A mausoleum of mumps: A case report

**DOI:** 10.1016/j.idcr.2024.e02006

**Published:** 2024-06-05

**Authors:** Benazir Begum R, Vivek Sanker, Nabeela Fatima, Vyshnav Rajagopal Menon, Lokesh Koumar Sivanandam, Tirth Dave

**Affiliations:** a JIPMER, Puducherry, India; b Team Erevnites, India; c Department of Neurosurgery, Trivandrum Medical College, Trivandrum, India; d Nizam Institute of Pharmacy, JNTUH, India; e Washington University of Health and Science, San Pedro, Belize; f Sri Lakshmi Narayana Institute of Medical Sciences, Puducherry, India; g Bukovinian State Medical University, Chernivtsi, Ukraine

**Keywords:** Retropharyngeal abscess, Pediatrics, Antibiotics, Mumps, Intra-oral drainage

## Abstract

**Introduction:**

Retropharyngeal abscess is a fatal infection that is uncommon, yet serious, especially in young children below 5 years. Oropharyngeal infections, in particular, can cause it as a complication of upper respiratory infections. They can also lead to respiratory depression and acute upper airway blockage and other complications.

**Case presentation:**

The unusual case of large retropharyngeal abscess in a 2-year-old child, secondary to mumps infection who presented to us with impending airway compromise is reported.

**Discussion:**

Retropharyngeal abscess secondary to mumps is a rare occurrence.The child initially had a bilateral parotid enlargement with fever and upper respiratory tract infection, which was diagnosed clinically as mumps by primary care physician and later confirmed by IgM antibody testing. The child was initially treated conservatively as the symptoms were mild at the beginning, however, the child worsened progressively and presented o our institute with acute retropharyngeal abscess in stridor. Emergency tracheostomy and intraoral abscess drainage were done under general anesthesia followed by appropriate intravenous antibiotics therapy.

**Conclusion:**

A dramatic recovery was observed following the treatment approach. Although it is rare to see such a large RPA in this antibiotic era, it is imperative to maintain a high index of suspicion.

## Introduction

Retropharyngeal abscess is a rare but serious fatal infection, particularly in children under the age of five. The buccopharyngeal fascia is located anteriorly, the prevertebral fascia is located posteriorly, and the carotid sheaths are located laterally to the pharynx to define the retropharyngeal space. It extends inferiorly to the mediastinum and superiorly to the base of the skull.

In adults, the retropharyngeal abscess is primarily due to foreign bodies, dental infections, instrumental procedures (laryngoscopy, endotracheal intubation, feeding tube placement, etc.), or trauma. It can also be secondary to upper respiratory infections specifically oropharyngeal infections [1]. Retropharyngeal abscesses can cause complications such as septicemia, mediastinitis, aspiration pneumonia, empyema, and significant vascular consequences such as internal jugular vein thrombosis and internal carotid artery erosion. They can also cause acute upper airway obstruction and respiratory depression [2].

Males are more likely to develop retropharyngeal abscesses than females, with a reported male prevalence of 53–55 %. The clinical manifestation is frequently sneaky, and the diagnosis may be challenging. Adults typically have a sore throat, fever, dysphagia, odynophagia, neck pain, and dyspnea as the main symptoms. Retropharyngeal abscess patients occasionally exhibit symptoms of airway obstruction, but not always. The most typical physical symptoms include cervical adenopathy, nuchal stiffness, posterior pharyngeal edema, drooling, and stridor. Retropharyngeal abscesses have diverse and non-specific clinical signs, making a clinical diagnosis challenging. In other cases of immunological suppression, such as diabetes, etc, the symptoms of infection may not be present [3].

In order to obtain the best outcomes, retropharyngeal abscesses necessitate early diagnosis and care, which commonly entail surgical draining. But there is still debate regarding when a surgical operation should be performed. Numerous organisms, such as aerobic organisms (beta-hemolytic Streptococci and Staphylococcus aureus), anaerobic organisms (species of Bacteroides and Veillonella), or Gram-negative organisms, can result in abscesses in this area (Haemophilus parainfluenzae and Bartonella henselae) [3].

These infections are now less frequent thanks to the early identification and the widespread use of antibiotics. Antibiotic therapy (often triple intravenous antibiotics: Co-amoxiclav, Aminoglycoside, and Imidazole) may not be sufficient in cases of nonspecific retropharyngeal abscess and the majority of papers suggest combining it with surgical draining of the collection. It is debatable when the drainage should be made, as mentioned earlier. Some literature suggests performing surgical drainage and local antibiotic injection simultaneously. Comorbidity treatment is a vital approach in management [3]. The aim of this case report is to discuss key aspects of the unusual presentation of large retropharyngeal abscess along with the review of literature.

## Case presentation

A 2-year-old Asian child belonging to a low socioeconomic class was referred by a local health physician to our center with 2 week's history of fever, upper respiratory tract infection (URI), bilateral neck swelling, difficulty in breathing and swallowing along with drooling of saliva. She was immunized up till date and had no known comorbidities. On probing history, the symptoms initially started with bilateral parotid swelling which was diagnosed in a private clinic as mumps based on the clinical features and serum testing for IgM antibody. The child received conservative management with analgesics, ensuring adequate hydration and isolation.This case report has been reported in line with the SCARE criteria [4].

When the child had persistent URI, a chest x-ray was taken which was normal and was treated as croup with nebulization. When the symptoms worsened to noisy breathing and extended neck posture the child was referred to our institute for further management in view of potential airway compromise.

**Fig. 1:**
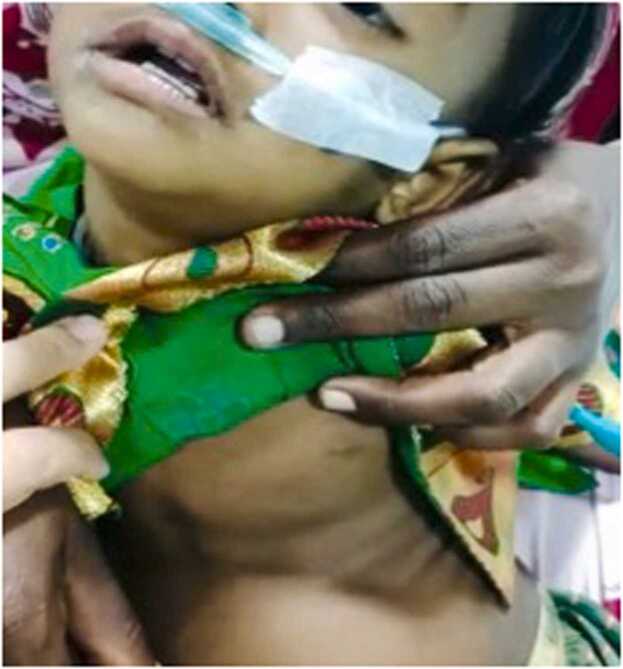
Shows drowsy child with severe intercostal and subcostal retraction.

On presentation, the child was drowsy and febrile. Her weight was 6.5 kg and her height was 78 cm (both below the 3rd percentile). There was tachycardia and tachypnea with severe subcostal and intercostal retraction (Fig. 1). Mild turbulence over the airway was noted but Saturation was maintained at around 90 % on room air. The child kept her neck extended, with restriction of any passive neck movement. Mouth opening with drooling of saliva was present. There were multiple bilateral levels II, III and IV enlarged cervical lymph nodes with no signs of inflammation. No obvious swelling is palpable on the neck. The carotid pulse was palpable and normal.

**Fig. 2:**
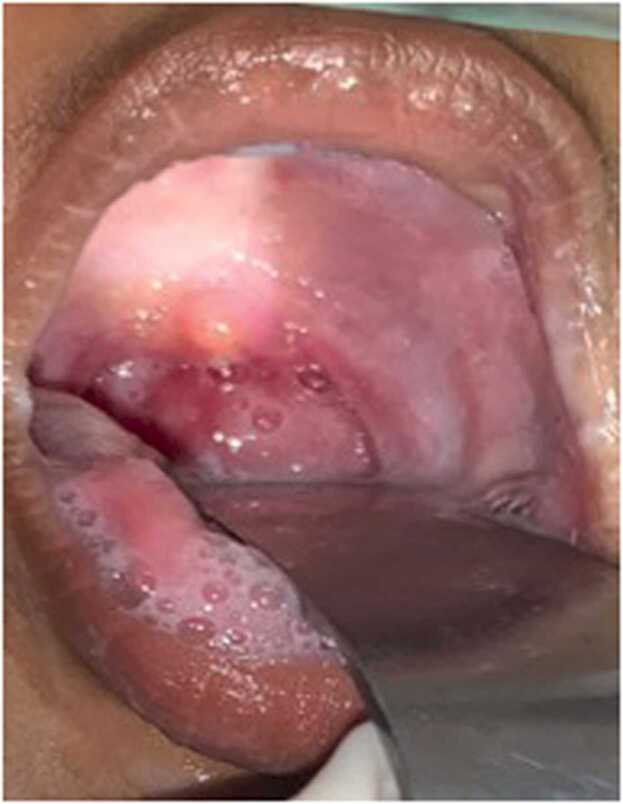
Oropharyngeal examination showing a bulge in the posterior pharyngeal wall predominantly to the left with salivary pooling.

Oropharyngeal examination showed a bulge in the left side of the posterior pharyngeal wall with congestion including the soft palate, uvula, and bilateral tonsillar region (Fig. 2). On otological examination, the bilateral tympanic membrane remained intact. The rest of the physical examination was normal. A clinical diagnosis of “*Acute retropharyngeal abscess with impending stridor”* was made.

**Fig. 3:**
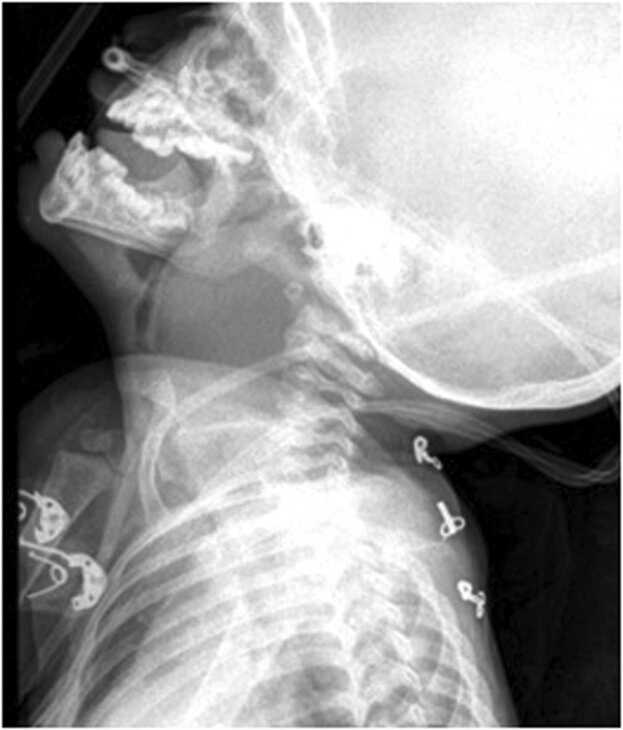
Plain lateral neck x-ray-reveals widening of soft tissue with anterior airway displacement. At the level of C2- 40 mm and at C6- 35 mm soft tissue widening is noted with extension of soft tissue shadow to the mid thoracic level.

Further investigations, including complete blood count, electrolytes, renal function test, and C-reactive protein test, were within normal ranges. Blood culture was sent during admission and empirically started on intravenous amoxy-clavulanic acid and metronidazole for gram-positive, gram-negative, and anaerobic coverage. X-ray soft tissue neck–lateral view and chest x-ray is taken bedside, which showed a large RPA (Fig. 3). Blood culture sensitivity was sterile after 48 h of incubation period. The child was not stable enough to shift for CECT or ultrasound, hence it couldn’t be performed.

After the emergency surgeon-anesthetist and panel of discussions, the primary aim was to secure the airway. The child was initially intubated with a relatively smaller size endotracheal tube (size 3.5). Emergency tracheostomy was deemed imperative, as there was a potential risk of aspiration postoperatively. Oblique tracheal incision done over 2nd tracheal ring with maturation suture taken on both sides, size 3.5 mm portex cuffed tracheostomy tube inserted. The free end of the sutures was attached to the chest.

**Fig. 4:**
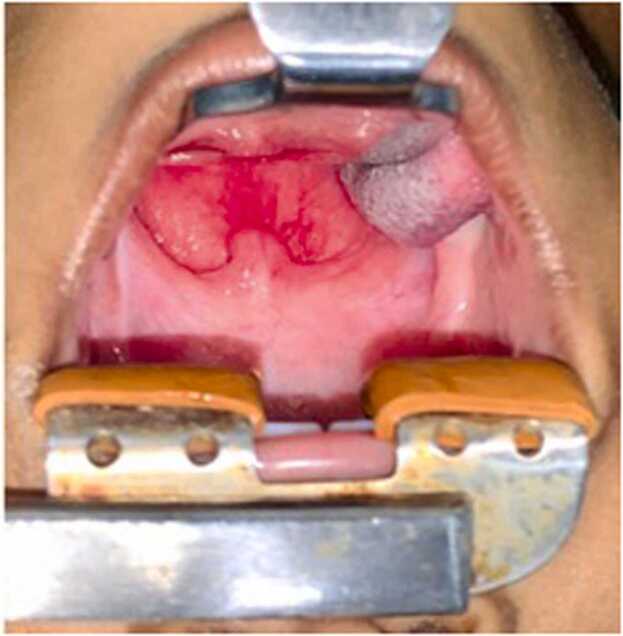
Boyle Davis mouth gag with tongue blade inserted. Bulge is noted in the posterior pharyngeal wall with maximum propensity to left. Uvula pushed anteriorly.

**Fig. 5:**
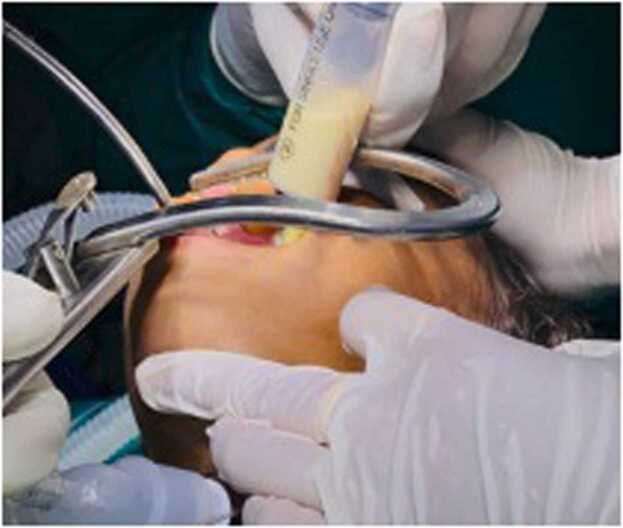
Intra-oral aspiration of purulent pus from the point of maximum bulge in posterior pharyngeal wall.

**Fig. 6:**
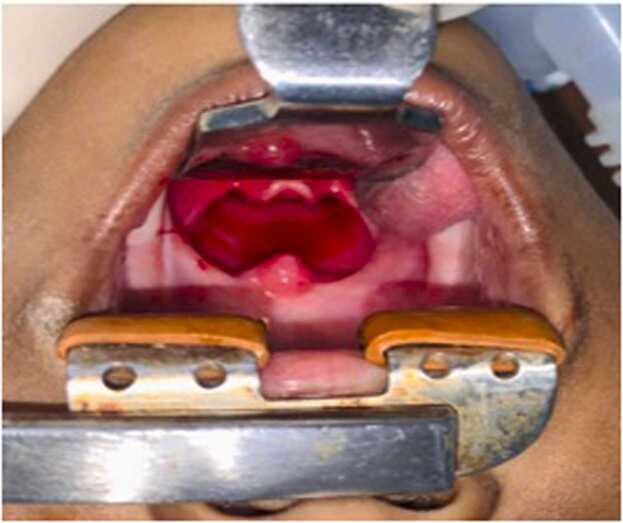
Oropharynx after intra-oral abscess drainage- significant reduction in posterior pharyngeal bulge noted in comparison to *figure-4* with increased room in oropharynx and visualization of epiglottis.

The child was placed in Trendelenburg position. Boyle- Davis mouth gag was inserted and a posterior pharyngeal wall bulge was noted. Epiglottis and vallecula could not be appreciated well, because of the pressure effect (Fig. 4). Intra-oral aspiration was done using a 50 ml syringe- around 20 ml of pus aspirated (Fig. 5). An incision was given at the point of the maximum bulge and around 30 ml of pus was drained and pus was sent for Gram stain and culture sensitivity. Bulge was reduced significantly; epiglottis could be visualized (Fig. 6). A feeding tube was placed at the end of the procedure. The child was kept under PICU observation postoperatively.

**Fig. 7:**
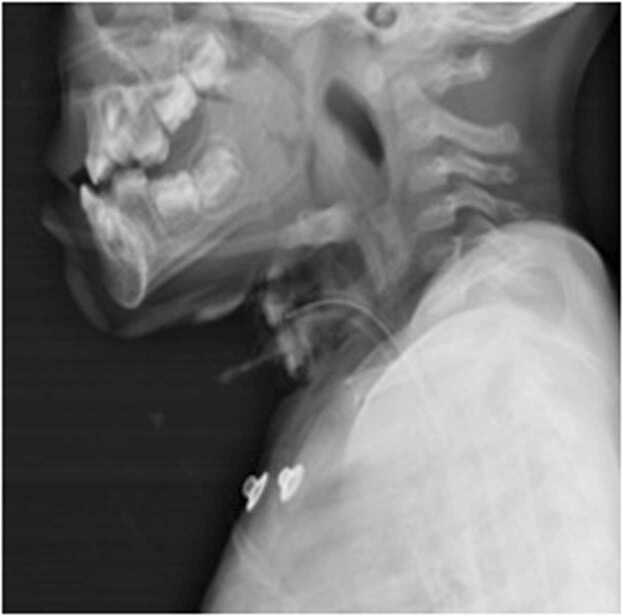
Post-operative lateral neck x-ray – remarkable reduction of soft tissue widening noted in comparison to pre-operative x-ray (Fig. 3), with Tracheostomy tube in position.

Postoperatively, a lateral neck x-ray was taken – a significant reduction in bulge was noted and the tracheostomy tube position was confirmed (Fig. 7). Intravenous antibiotics continued for 7 days. Gram stain from the microbial swab showed presence of pus cells and no organism. Blood and intraoperative pus culture sensitivity- reported sterile after 48 h of the aerobic incubation period. On Post-operative day 7- a trial block of the tracheostomy tube was done. The child tolerated the trial block for more than 24 h, hence de-cannulation and strapping were done. The child was discharged on postoperative day 8 and on follow up, a week and a month later, child was able to swallow and breathe comfortably. On examination, posterior pharyngeal wall was normal and there were no palpable lymph nodes.

## Discussion

An acute retropharyngeal abscess (RPA) is a rare clinical entity. Suppuration of rouviere’s lymph node in the retropharyngeal space is the most common cause of RPA in children. The other causes can be due to untreated foreign body impaction, malignancies or as an extension deep neck abscess in adjacent spaces. In our case, child presented with bilateral parotitis which was diagnosed as mumps confirming with a serum IgM antibody testing, and was treated conservatively by the primary care physician. However, the child’s symptoms did not improve and progressed to develop retropharyngeal abscess, probably due to under-treatment, low immunity of the child or increased virulence of the organism.

The retropharyngeal space (the space of gillete) lies behind the pharynx between the buccopharyngeal fascia and extends from the base of the skull to the tracheal bifurcation. Retropharyngeal space communicates laterally with Para pharyngeal space (PPS) that steadily increases the morbidity of the disease [5]. Our patient presented with neck stiffness, fever and labored. As the patient presented to the pediatrics emergency with respiratory distress and X-ray soft tissue of neck revealed a large retropharyngeal abscess on the lateral view, the child was immediately taken to the operating theater to secure the airway and drain the abscess; hence, further CECT-neck and assessment of extension of abscess to the adjacent spaces could not be made. However, considering the width of the retropharyngeal space (C2- 40 mm and at C6- 35 mm) and mediastinal extension, the possibility of abscess spread to PPS could not be absolutely ruled out. The main aim of the management of RPA with impending airway should be securing the airway rather to evaluate for the disease extension.

Dental infection and upper airway infection remain the two most common etiologies for RPA in adults and children, respectively [6] Mumps is not very uncommon in children. It presents as bilateral parotitis with fever and upper respiratory tract infection. Severe complications like meningoencephalitis, cerebellar ataxia, arthritis, orchitis, transverse myelitis, deafness, and pre-sternal edema have been reported in various studies [7]. The lymphatics from the salivary glands, posterior pharyngeal wall, and nasopharynx drain to retropharyngeal nodes. The inadequately treated mumps with inflamed salivary glands can cause suppuration of retropharyngeal nodes and ignite the event of RPA in mumps.Such association of RPA with mumps is rarely reported worldwide.RPA is more common in children with low socio-economic status [8]. The threshold should be low to start antibiotics in such high-risk patients who present patients with neck swelling, and patients with respiratory difficulty [9].

The first line of investigation is lateral soft tissue neck x-ray. However, it has a false-negative rate of 33 % [10]. Hence, the normal lateral neck x-ray need not always rule out the absence of RPA. CECT of the neck and thorax is useful to differentiate abscesses from those with retropharyngeal cellulitis with additional information about the mediastinal involvement [11], [12]. The use of CECT, should be limited to the patients whose vitals are stable without an airway threat.

A high dose of broad-spectrum antibiotic should be started empirically as it controls the further spread and at times obviate the need for surgical intervention, particularly in small abscesses less than 25 mm [13]. Initiate early start of empirical parenteral antibiotic therapy where clinical suspicion of retropharyngeal cellulitis/abscess is high [14], [15]. The decision to initiate surgical drainage depends on the patient’s clinical status and the accessibility of the abscess [16]. Since the airway was at threat in our patient, trial of conservative management had no role and warranted an immediate surgical intervention with concurrent antibiotic therapy.

Intubation for surgery depends on the status of the airway. In RPA, the chances of successful intubation is low as the airway can be pushed anteriorly, edematous and risk of perioperative aspiration, in such situations tracheostomy is deemed necessary. Though the airway in our child is pushed anteriorly, a trial of intubation with smaller size endotracheal tube (3.5 mm) was given to aid in securing airway and sedate the child prior to tracheostomy with back-up laryngeal mask airway. Tracheostomy was done post intubation, airway was secured and position of tracheostomy tube was confirmed with auscultation and ETCO2. In our patient, 20 ml of pus drained intra-orally following a wide bore aspiration. Various case reports and series, support intraoral drainage of an abscess in large RPA unless there is involvement or extension lateral to great vessels [17]. Post-operatively, the child was observed in pediatric ICU with nasogastric feed for 72 h followed by clear fluid orally. Early start of oral clear liquid diet is considered and an advanced slowly to a soft diet over a period of several days so as to allow appropriate healing of the surgical site [18].

## Conclusion

RPA is a potentially lethal condition that can result from an episode of upper respiratory infection or a straightforward infection in these regions. The disease's morbidity can gradually rise as a result of infection spreading from the retropharyngeal space to PPS. Consequently, it is crucial to start treatment as soon as a problem is identified.Since it occasionally eliminates the need for surgical intervention, especially in cases with tiny abscesses, a high dose of antibiotic should be begun empirically. Retropharyngeal cellulitis may proceed to an abscess, although early medical intervention may prevent this from happening. It is crucial to keep a high index of suspicion even though it is uncommon to observe such a huge RPA in this antimicrobial era.

## Ethical approval

Not required.

## Sources of Funding

None.

## Author contribution

All authors contributed equally in drafting, revising and finalizing the manuscript for submission.

## Conflicts of Interests

None declared.

## Consent

Written informed consent was obtained from the patient for publication of this case report and accompanying images. A copy of the written consent is available for review by the Editor-in-Chief of the journal on request.

## Author Contribution

All authors contributed equally.

## Provenance and peer review

Not commissioned, externally peer reviewed.

## CRediT authorship contribution statement

**Lokesh Koumar Sivanandam:** Writing – original draft, Writing – review & editing. **Tirth Dave:** Project administration, Supervision, Writing – original draft, Writing – review & editing. **Benazir Begum R:** Conceptualization, Data curation, Writing – original draft, Writing – review & editing. **Vivek Sanker:** Conceptualization, Data curation, Writing – original draft, Writing – review & editing. **Nabeela Fatima:** Conceptualization, Data curation, Writing – original draft, Writing – review & editing. **Vyshnav Rajagopal Menon:** Writing – original draft, Writing – review & editing.

## Declaration of Competing Interest

Authors declare no conflict of interests.

## References

[1] Goldenberg D., Golz A., Joachims H.Z. (1997). Retropharyngeal abscess: a clinical review. J Laryngol Otol.

[2] Coulthard M., Isaacs D. (1991). Retropharyngeal abscess. Arch Dis Child.

[3] Harkani A., Hassani R., Ziad T., Aderdour L., Nouri H., Rochdi Y. (2011). Retropharyngeal abscess in adults: five case reports and review of the literature. Sci. World J..

[4] Agha R.A., Franchi T., Sohrabi C., Mathew G. (2020). Lignedirectrice SCARE 2020: mise à jour des lignes directrices du rapport sur les caschirurgicaux de consensus (SCARE). Int J Surg.

[5] Mydam J., Thiagarajan P. (2009). A nine month old child with retropharyngeal abscess secondary to mastoid abscess presenting as torticollis: a case report. Cases J.

[6] Wang L.F., Tai C.F., Kuo W.R., Chien C.Y. (2010). Predisposing factors of complicated deep neck infections: 12-year experience at a single institution. J Otolaryngol–Head Neck Surg.

[7] Nussinovitch M., Volovitz B., Varsano I. (1995). Complications of mumps requiring hospitalization in children. Eur J Pediatr.

[8] Grisaru-Soen G., Komisar O., Aizenstein O., Soudack M., Schwartz D., Paret G. (2010). Retropharyngeal and parapharyngeal abscess in children—epidemiology, clinical features and treatment. Int J Pediatr Otorhinolaryngol.

[9] Wang L.F., Kuo W.R., Tsai S.M., Huang K.J. (2003). Characterizations of life-threatening deep cervical space infections: a review of one hundred ninety-six cases. Am J Otolaryngol.

[10] Uzomefuna V., Glynn F., Mackle T., Russell J. (2010). Atypical locations of retropharyngeal abscess: beware of the normal lateral soft tissue neck X-ray. Int J Pediatr Otorhinolaryngol.

[11] Craig F.W., Schunk J.E. (2003). Retropharyngeal abscess in children: clinical presentation, utility of imaging, and current management. Pediatrics.

[12] Lautermann J., Lehnerdt G., Beiderlinden M., Sudhoff H. (2005). Infections of the deep neck spaces with accompanying mediastinitis. Laryngo-Rhino-Otologie.

[13] Wong D.K., Brown C., Mills N., Spielmann P., Neeff M. (2012). To drain or not to drain–management of pediatric deep neck abscesses: a case–control study. Int J Pediatr Otorhinolaryngol.

[14] Patel Vijay A, Elluru Ravindhra G. Pediatric retropharyngeal abscess medication updated: Mar 08, 2021. Pediatric retropharyngeal abscess medication: penicillins, amino, glycopeptides, lincosamides, cephalosporins, 2nd generation, penicillins, extended-spectrum (medscape.com).

[15] Gaglani M.J., Edwards M.S. (1995). Clinical indicators of childhood retropharyngeal abscess. Am J Emerg Med.

[16] Fédérici S., Silva C., Maréchal C., Laporte E., Sévely A., Grouteau E. (2009). Retro-and parapharyngeal infections: standardization of their management. Arch Pediatr: Organe Soc Pediatr.

[17] Kirse D.J., Roberson D.W. (2001). Surgical management of retropharyngeal space infections in children. Laryngoscope.

[18] Patel Vijay A, Elluru Ravindhra G. Pediatric retropharyngeal abscess treatment & management updated: Mar 08, 2021. Pediatric retropharyngeal abscess treatment & management: approach considerations, medical care, surgical care (medscape.com).

